# Supplementary material for: Investigation on the Adsorption-Interaction Mechanism of Pb(II) at Surface of Silk Fibroin Protein-Derived Hybrid Nanoflower Adsorbent
Source: Materials (Basel). 2020 Mar 9;13(5):1241. doi: 10.3390/ma13051241 (PMC7085063; doi:10.3390/ma13051241)
Supplement: Supplementary file 1 [file materials-13-01241-s001.docx]

Article

Investigation on the Adsorption-Interaction Mechanism of Pb(II) at Surface of Silk Fibroin Protein-Derived Hybrid Nanoflower Adsorbent

Xiang Li ^1^, Yan Xiong ^1,^*, Ming Duan ^1,^*, Haiqin Wan ^2^, Jun Li ^1^, Can Zhang ^1^, Sha Qin ^1^, Shenwen Fang ^1^, and Run Zhang ^3^

^1^ School of Chemistry and Chemical Engineering, Southwest Petroleum University, Chengdu, 610500, China; 201721000245@stu.swpu.edu.cn (X.L.); 201821000253@stu.swpu.edu.cn (J.L.); 201922000213@stu.swpu.edu.cn (C.Z.); 201921000230@stu.swpu.edu.cn (S.Q.); mduana124@swpu.edu.cn (S.F.)

^2^ State Key Laboratory of Pollution Control and Resource Reuse, Jiangsu Key Laboratory of Vehicle Emissions Control, School of the Environment, Nanjing University, Nanjing 210023, China; wanhq@nju.edu.cn

^3^ Australian Institute for Bioengineering and Nanotechnology, AIBN, The University of Queensland, St Lucia, QLD 4072, Australia; r.zhang@uq.edu.au

***** Correspondence: 201031010013@swpu.edu.cn (Y.X.); mduan@swpu.edu.cn (M.D.)

Received: 18 February 2020; Accepted: 06 March 2020; Published: date


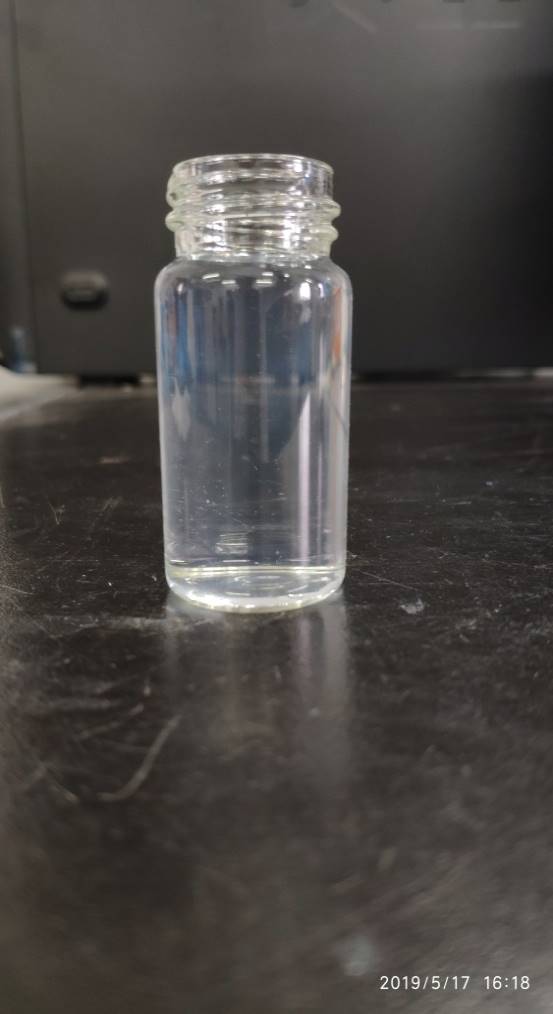


**0 min**


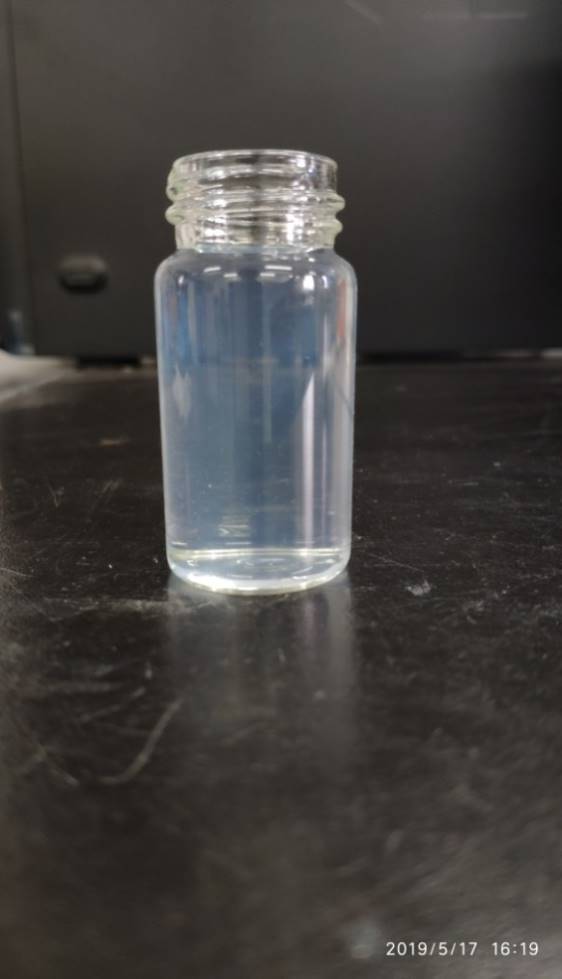


**10 min**

**Figure S1.** Photography of solution change at reaction times of 0 min and 10 min.

**Figure S2.** Diameter distribution of products from 0 to 24 h.

**Figure S3.** (**a**) The surface zeta potential measurement of SF@Cu-NFs at pH=4.0–9.0; (**b**) Adsorption capacity of the prepared SF@Cu-NFs for Pb(II) at pH=4.0–9.0.

**Figure S4.** The adsorption isotherms of Pb(II) fitting by (**a**) Langmuir model, (**b**) Freundlich model and (**c**)Temkin model.

**Figure S5.** Evaluation of SF@Cu-NFs adsorption selectivity for (i) Pb(II), (ii) Cd(II) and (iii) Ni(II). Insets are HMIs solution after different adsorption of 0, 5, 20, 40 and 90 min. The chromogenic reagent for Pb(II) and Cd(II) are DTZ and for Ni(II) is DMG.

**Figure S6.** XRD spectra of SF@Cu-NFs before (spectrum i) and after (spectrum ii) Pb(II) adsorption.

**Table S1.** The related EDS data of SF@Cu-NFs.

| **Element** | **Line Type** | **Atomic%** | **Wt.%** |
| --- | --- | --- | --- |
| C | K series | 44.38 | 27.18 |
| O | K series | 40.70 | 33.21 |
| P | K series | 3.69 | 5.83 |
| Cl | K series | 1.80 | 3.26 |
| Cu | K series | 9.42 | 30.52 |
| Total: |  |  | 100.00 |

**Table S2.** Comparison maximum capacity of SF@Cu-NFs products in this work and other adsorbents for Pb(II) adsorption.

| **Adsorbents** | **pH** | **Temperature (°C)** | $\boldsymbol{Q}_{\boldsymbol{max}}$ **(mg g^−1^)** | **References** |
| --- | --- | --- | --- | --- |
| Algal Chlorella | 6.0 | 25 | 635.80 | [[8](#_ENREF_8)] |
| Polyampholyte hydrogel | 5.0 | 40 | 216.10 | [[60](#_ENREF_60)] |
| Nanohydroxyapatite powder | 3.0 | 23 | 200.00 | [[61](#_ENREF_61)] |
| jute/polyacrylic acid hydrogel | 5.0 | 40 | 542.90 | [[62](#_ENREF_62)] |
| MoS_2_ microrods/C | 5.0 | 25 | 303.04 | [[63](#_ENREF_63)] |
| GO/CNTs membrane | 5.0 | 25 | 98.00 | [[64](#_ENREF_64)] |
| BSA-Au hybrid | 7.0 | 25 | 270.00 | [[65](#_ENREF_65)] |
| Natural Artemia CS | 6.2 | 20 | 320.00 | [[66](#_ENREF_66)] |
| SF@Cu-NFs | 5.0 | 25 | 1908.39 | This work |

**Table S3.** Thermodynamic data for Pb(II) adsorption by SF@Cu-NFs at different temperatures

| $\boldsymbol{C}_{\boldsymbol{0}}$ **(mg L^−1^)** | **T (K)** | $\boldsymbol{K}_{\mathbf{T}}^{\boldsymbol{\theta}}$ | ${\boldsymbol{\Delta}_{\boldsymbol{r}}\boldsymbol{G}}_{\mathbf{m(T)}}^{\boldsymbol{\theta}}$**(kJ mol^−1^)** | ${\boldsymbol{\Delta}_{\boldsymbol{r}}\boldsymbol{H}}_{\mathbf{m}}^{\boldsymbol{\theta}}$**(kJ mol^−1^)** | ${\boldsymbol{\Delta}_{\boldsymbol{r}}\boldsymbol{S}}_{\mathbf{m}}^{\boldsymbol{\theta}}$**(J mol^−1^ K^−1^)** | $\boldsymbol{R}^{\boldsymbol{2}}$ |
| --- | --- | --- | --- | --- | --- | --- |
| 100 | 298 | 38.89 | −9.07 | −64.30 | −184.57 | 0.99 |
|  | 308 | 21.32 | −7.84 |  |  |  |
|  | 328 | 3.78 | −3.63 |  |  |  |
| 500 | 298 | 6.08 | −4.47 | −17.91 | −45.06 | 0.99 |
|  | 308 | 4.83 | −4.03 |  |  |  |
|  | 328 | 3.14 | −3.12 |  |  |  |

| 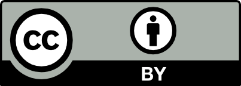 | © 2020 by the authors. Submitted for possible open access publication under the terms and conditions of the Creative Commons Attribution (CC BY) license (http://creativecommons.org/licenses/by/4.0/). |
| --- | --- |
